# Biochemical and Biophysical Characterization of the Enolase from* Helicobacter pylori*

**DOI:** 10.1155/2018/9538193

**Published:** 2018-12-17

**Authors:** María de J. López-López, Isabel C. Rodríguez-Luna, Edgar E. Lara-Ramírez, Marisol López-Hidalgo, Claudia G. Benítez-Cardoza, Xianwu Guo

**Affiliations:** ^1^Centro de Biotecnología Genómica-Instituto Politécnico Nacional, Centro de Biotecnología Genómica. Boulevard del Maestro S/N Esquina Elías Piña, Colonia Narciso Mendoza, 88710, Cd. Reynosa Tamaulipas, Mexico; ^2^Unidad de Investigación Biomédica de Zacatecas, Instituto Mexicano del Seguro Social (IMSS), 98000, Zacatecas, Mexico; ^3^Laboratorio de Investigación Bioquímica, ENMyH-Instituto Politécnico Nacional, Guillermo Massieu Helguera No. 239, La Escalera Ticoman, 07320, Ciudad de México, Mexico

## Abstract

Enolase, which catalyses the conversion of 2-phospho-D-glycerate to phosphoenolpyruvate, is an important enzyme in the classic glycolysis pathway in cells. Enolase is highly conserved in organisms from bacteria to humans, indicating its importance in cells. Thus, enolase is a good target for developing new drugs. In the last decade, new functions of this enzyme have been found.* Helicobacter pylori* is a common human pathogen that causes gastric diseases and even gastric cancer. In this study, the sequence of* H. pylori* enolase (HpEno) was analysed; the conservation (at least partial) of binding sites for cofactor, plasminogen, and host extracellular RNA, as well as catalytic site, indicates that HpEno should be capable of performing the functions. Recombinant HpEno was overexpressed and purified from* E. coli*. Compared to the enolases from other species, HpEno had similar characteristics for its secondary structure. The temperature-induced profiles indicate that HpEno is quite stable to temperature, compared to other homologs. Regarding the kinetics of the unfolding reaction, we found that the activation enthalpy associated with the thermal unfolding reaction is equivalent to the reported activation enthalpy for yeast enolase, indicating a similar scaffold and kinetic stability. Although a wide range of experimental conditions were assayed, it was not possible to detect any enzymatic activity of HpEno. To prove the lack of activity, still a much wider range of experiments should be carried out.

## 1. Introduction

Enolase is an enzyme that catalyses dehydration of 2-phospho-D-glycerate into phosphoenolpyruvate during glycolysis and its reverse reaction in gluconeogenesis, which is often vital for cellular function [1]. This enzyme requires Mg^2+^ for catalysis and can also be activated by other divalent cations [2]. Enolase is a conserved protein with catalytic properties that are similar among divergent organisms. The ubiquitous presence of the enzyme and the sequence homology between enolases from extant organisms belonging to different phyla indicate that the enolase gene was already present in their common ancestor and that this gene became diversified by the speciation of organisms or by gene duplication within species [3]. Furthermore, enolase has been described as a moonlighting protein, performing a variety of functions in several biological and pathophysiological processes within a set of subcellular compartments [4–6]. For example, the enolase from eukaryotic cells is involved in the regulation of cell morphology through the regulation of cytoskeletal filament dynamics [7]. Mammalian enolase participates in the transcriptional regulation of genes involved in cell proliferation [8], and bacterial enolase has been described as an important virulence factor due to its binding to plasminogen [9–11] and as a key component of the degradosome, where its roles are still poorly understood [12].

Enolase has been frequently reported as homodimer in most organisms or as an octamer in some thermophilic bacteria [13–18]. The structure of the monomer consists of an eightfold *β*/*α* barrel domain preceded by an N-terminal *α*+ *β* domain [19]. The divergence of the protein sequences, structures, and functions reveals the intrinsic potential of its folding pattern.

*Helicobacter pylori* is a gram-negative bacterium that infects the human upper gastrointestinal tract of over 50% of the population worldwide [20]. Treatment to eradicate the infection consists of combining two or three antibiotics with one proton pump inhibitor. However, reports are mounting for cases of resistant strains leading to ineffective therapy [21]. This finding implies that new strategies for treating this infection are necessary.* H. pylori* has a reduced genome size (1.7 Mb), and many proteins have been lost in its evolutionary history, and the extant proteins can change their functions. In our laboratory, the enolase from* H. pylori* (HpEno) was pulled down with the protein MreB, an actin-like protein, indicating that HpEno can exist in a complex with MreB [22]. Thus, the enolase protein may have different structural and functional characteristics in this bacterium. Here, for the first time, we present the production and purification of enolase from this pathogen and analysed some of its structural, biochemical, and biophysical characteristics, which could shed light on the structure-function relationship of this protein in* H. Pylori* [23].

## 2. Material and Methods

### 2.1. Cloning and Overexpression

The gene encoding enolase was amplified from* H. pylori* strain 26695 by polymerase chain reaction (PCR). Briefly, the primers were designed according to the sequence in the NCBI database with accession number CP003904.1, resulting in the forward primer 5′GGCCATATGATGCTAACCATTAAAGATATTC3′ and the reverse primer 5′AATACTCGAGCTAGCCATG CTTAAACAACTC3′ with a NdeI or XhoI restriction site, respectively (underlined). The PCR product was cloned into the vector pGEM-T Easy (Promega, USA). Subsequently, the insert digested with NdeI/XhoI was moved to a modified version of the expression plasmid pET-19b-mod (Harvard University, USA), which can express a protein with an N-terminal 10-histidine tag and contains a PreScission protease (PSP) cleavage site, producing a new plasmid, pET-19b-mod-HpEno, which was then transformed into* Escherichia coli* strain DH5*α*. The insertion was confirmed by PCR using the T7 promoter primer and pUC/M13 reverse primer and by sequencing with the ABI Prism AB3130 (Applied Biosystems, USA) sequencer.

Several strains of* E. coli* and several incubation conditions were evaluated for overexpression of HpEno. The optimum expression was achieved by incubating strain Rosetta-gami at 37°C. A single colony was picked and inoculated in 10 ml of 2×YT medium supplemented with ampicillin 100 *μ*gmL^−1^ and chloramphenicol 34 *μ*gmL^−1^ and grown overnight at 37°C with shaking at 200 rpm. Afterwards, this culture was diluted 1:100 in either LB or 2×YT medium supplemented with the same antibiotics, and the cells were grown at 200 rpm to an optical density (OD_600_) of 0.6. Overproduction of the recombinant protein was induced by adding 1 mM isopropyl *β*-D-1-thiogalactopyranoside (IPTG) [24] for four hours after addition of IPTG, to get a final concentration of 1 mM. The cells were pelleted by centrifugation.

### 2.2. Protein Purification

The pellet was treated as described previously [25] (see supplementary materials: appendix 1). The purification was performed by affinity chromatography in an Econo-Column (Bio-Rad Laboratories, USA) packed with Ni-NTA resin (QIAGEN, Germany). The supernatants from the previous step were loaded onto a column equilibrated with binding buffer (50 mM Tris-HCl (pH 8.0), 300 mM KCl, 10 mM imidazole, and 8 M urea. Some purification trials were carried out substituting 8M urea for 6M guanidinium chloride. The column was washed with ten-column volumes of wash buffer (50 mM Tris-HCl (pH 8.0), 300 mM KCl, and 50 mM imidazole with a gradient of decreasing urea concentration from 8 to 0 M, or guanidinium chloride from 6M to 0M), and finally, the bound protein was eluted with five-column volumes of elution buffer (50 mM Tris-HCl (pH 8.0), 300 mM KCl, and 300 mM imidazole). The His-tag was removed from enolase with PSP, following the manufacturer's instructions. PSP was eliminated by a glutathione sepharose (GE Healthcare, USA) column, following the manufacturer's instructions. The uncleaved* H. pylori* enolase in the protein solution was separated by Ni-NTA resin. The sample homogeneity was confirmed by an SDS-PAGE gel stained with Coomassie blue. The protein concentration was estimated spectrophotometrically at 280 nm, using an extinction coefficient, *ε*_280  nm_, of 30495 M^−1^ cm^−1^ [26]. All reactants were of analytical grade, and the water used was distilled and deionized. In all cases, data are reported as the average of at least three independent experiments.

### 2.3. Activity Assays

The coupled reactions of rabbit muscle pyruvate kinase (PYK) and beef heart lactate dehydrogenase (LDH) were used to evaluate the enolase activity by following the decrease in NADH absorbance at 340 nm using a Scinco S-3100 UV-Vis spectrophotometer. Standard activity assays were performed as described earlier [27] (see details in the supplementary materials: appendix 1).

The activity was also assayed in a variety of experimental conditions, for example, by substituting triethanolamine/HCl buffer for either Tris-HCl, or tris-acetate, or HEPES at the same concentration and pH. Additionally, the concentration of each reactant was varied as follows: 2-PGA (1.9-125 mM), ADP (1.3 -2.5 mM), *β*-NADH (0.12-0.52mM), MgSO_4_ (0.05-25 mM), KCl (18.8-100 mM), PYK (7-23 U mL^−1^) and LDH (10- 33 U mL^−1^). Enolase from crude extracts, enolase purified with the His-tag, or cleaved recombinant enolase was added at final concentrations of 400 to 1800 ng mL^−1^. Positive control experiments were performed using 0.025 to 0.05 units of commercial enolase from* Saccharomyces cerevisiae* (ScEno) purchased from Sigma.

### 2.4. Far-UV Circular Dichroism

The secondary structure of recombinant HpEno was analysed by circular dichroism (CD) using a JASCO J-815 spectropolarimeter (Jasco Inc., Easton, MD) equipped with a PFD-425S Peltier-type cell holder for temperature control and magnetic stirring. CD spectra were recorded using 1 cm path length cells from 200 to 250 nm. Ellipticities are reported as the mean residue ellipticity, [*θ*]_MRW_.

### 2.5. Thermal Denaturation Transitions Monitored by Circular Dichroism

Thermal denaturation transitions were carried out as described before [28–31] (also see the methodological details and data analysis in the supplementary materials: appendix 1).

### 2.6. Fluorescence Spectra

Fluorescence spectra were obtained using an LS-55 Spectrofluorometer (Perkin–Elmer) equipped with a water-jacketed cell holder for temperature control. All the experiments were performed using cells with a path length of 1.0 cm at 25°C. The excitation wavelengths were 280 nm and 295 nm. Experiments were carried out in similar buffer conditions as for the far-UV circular dichroism assays.

### 2.7. Denaturation Kinetics

The kinetic curves of HpEno were obtained by recording changes in ellipticity at 220 nm, by the methods described previously [32, 33] (see the methodological details and data analysis in the supplementary materials: appendix 1).

### 2.8. Alignment of Sequences

Sequences of enolase from* H. pylori* and other validated bacterial and eukaryotic enolase sequences were obtained from the NCBI database. Multiple sequence alignment was performed with SeaView software [34], and the GeneDoc program was used to visualize the alignment. In this manuscript, we indicate the position of each residue in the sequence of each enolase and, for a common reference, also indicated the position of that same residue (in parenthesis) according to the sequence of* S. cerevisiae* enolase, as shown at the top of this alignment.

## 3. Results

### 3.1. Sequence Analysis of Functional Domains in HpEno

*H. pylori* enolase shares 46%, 53%, and 56% identity to yeast,* E. coli,* and* Xylella fastidiosa* enolase, respectively. In several organisms, it has been proved that enolase can perform several functions in addition to its innate glycolytic function, thus participating in several biological and pathophysiological processes. Furthermore, enolase has been found in thecytoplasmic and extracellular proteomes of* H. Pylori *[35, 36]. Therefore, we analysed the sequence of HpEno to find out whether this protein might be capable of performing enzymatic activity and other moonlighting functions reported previously for other enolases, as judged from the conservation of the corresponding functional motifs.

### 3.2. Catalytic and Metal-Binding Site

**Figure 1:**
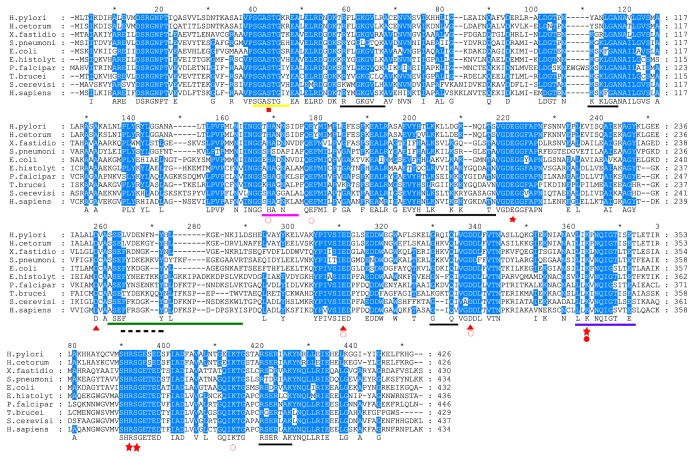
**Alignment of the amino acid sequences of HpEno with homologs from different biological species**. The species include* H. pylori *26695 (P48285)*, H. cetorum *(I0ELQ0)*, X. fastidiosa *(Q9PDT8)*, S. pneumoniae *(Q97QS2)*, E.coli *(P0A6P9)and* E. histolytica *(N9UZM6)*, P. falciparum *(Q27727)*, T. brucei *(Q38BV6)*, S. cerevisiae *(P00924), and human (*α*-enolase, P06733). The red signals under the letters represent rectangle, metal binding site reported only for human*α*-enolase; open circle, substrate binding sites; star, active site residues; triangle, metal binding sites; close circle, subtract binding site in inhibited form. The black lines indicate the sites for RNA-binding and the dashed lines regions for plasminogen binding in* S. pneumonia*. The yellow line corresponds to loop L1, pink line to loop L2, and green line to loop L3, blue line corresponds to signature of enolase.

The catalytic site of enolase is composed of seven residues (Table 1), [18, 37–39]. H^155^, E^164^, E^205^, K^338^, R^367^, S^368^, and K^389^ (in the* H. pylori* numbering system) [40] are all conserved in* H. pylori* enolase. Furthermore, for human *α*-enolase (hENO1), three loops involved in catalysis have been described, L1 (^37(37)^SGASTGIY^44(44)^), L2 (^157(159)^SHAGNKL^163(165)^), and L3 (^248(266)^ASEFYRDGKYDLDFNSPDDPSRYI^271(274)^) [37]. In HpEno, L1 and L2 have the sequences ^37(37)^SGASTGKR^44(44)^ and ^157(159)^THANNSI^163(165)^, respectively, while L3 (^245(266)^SSELVDENFNYHLKGENKIL^264(274)^) is not importantly conserved (Table 1). Substitutions in L1 or L2 have been considered attractive drug targets against the pathogens, such as trypanosomatid parasites [5]. L3 is poorly conserved in other active enolases, such as the* E. histolytica, S. cerevisiae,* and* S. pneumonia* enolases. In addition, enolase requires binding of a divalent cation (commonly Mg^2+^) as a cofactor to perform catalysis. The residues involved in Mg^2+^ binding reported for hENO1 and enolases from other species are E^293(296)^, D^245(247)^, D^318(321)^, and S^40(40)^ [1, 41–43]. All of these residues are conserved in* H. pylori* enolase, so this enzyme should be capable of binding divalent cations and performing the enzymatic reaction (Table 1, Figure 1).

### 3.3. Plasminogen-Binding Site

Enolases from several organisms have been found to bind plasminogen on the cell surface [4, 10, 44, 45]. Two plasminogen-binding sites have been identified [18, 45–48]. The “C-terminal site” is formed by two lysine residues (^433(435)^KK^434(436)^) at the C-terminal end. In the case of enolase from* H. pylori*, the C-terminus does not have two lysine residues; instead, KHG were found (Table 1, Figure 1). Therefore, the positive character of the side chains is conserved at the C-terminus. The second plasminogen-binding site, known as the “internal site”, is a nine-residue motif (^248(253)^FYDKERKVY^256(259)^) in the enolase sequence of* S.pneumoniae*. This motif is commonly composed of positively and negatively charged residues flanked by hydrophobic residues. In enolase from* H. pylori*, the site could be composed of ^248^LVDENFNY^255^, implying that the putative plasminogen-binding site of HpEno might display only the characteristic acidic residues but not the basic ones. Thus, whether HpEno is capable of binding plasminogen remains unclear.

### 3.4. Host Extracellular RNA- (exRNA-) Binding Site

Extracellular enolase has been recognized as a novel host-derived exRNA-binding protein on the surface of* S. pneumoniae* cells. The binding motifs are ^59^RYGGLGTQK67, ^104^KGKLGA^109^, ^188^HALKKILKSRGLETA^202^, ^312^GKKVQL^317^, ^401^RTDRIAK^408^, and ^432^LKK^434^ [49]. Several amino acid residues of these motifs, at least in terms of the characteristics of their side chains, are conserved in HpEno (Table 1), except for the second motif, which has two lysine residues that are not found in HpEno. Therefore, although this possibility still has to be experimentally analysed and confirmed, HpEno might interact with host-derived exRNA, thus contributing to the colonization and dissemination of bacteria [49].

### 3.5. Overexpression and Purification of Recombinant HpEno

**Figure 2:**
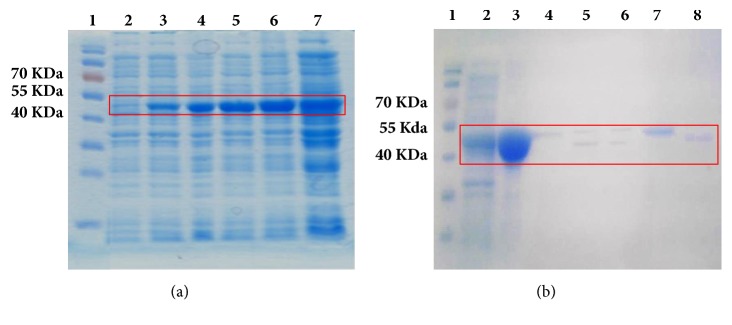
**SDS-PAGE electrophoresis of expression and purification of HpEno**. (a) Overexpression of HpEno in* E.coli *strain Rosetta-gami, lane 1: prestained protein marker (7-175 kDa), lane 2: induction with IPTG (T_o_), lane 3: induction for 1 hour (T_1_), lane 4: induction for 2 hours (T_2_), lane 5: induction for 3 hours (T_3_), lane 6: induction for 4 hours (T_4_), and lane 7: induction overnight (T_O∖N_). (b) Affinity chromatography and His-tag removal, lane 1: prestained protein marker (7-175 kDa), lane 2: overexpression of HpEno, lane 3: solubilization of inclusion bodies, lane 4: flow through, lane 5: his-tag cut with PSP after 18 hr, lane 6: his-tag cut with PSP after 24 hr, lane 7: enolase no-cleaved, abd lane 8: HpEno cleaved.

Several strains of* E. coli* and several incubation conditions were evaluated for the overexpression of HpEno. For the selection of the best host for protein production, several strains of* E. coli*, such as BL21 (DE3) pLysS, BL21 (DE3), and Rosetta-gami, were evaluated with the plasmid pET-19b-mod-HpEno. The Rosetta-gami strain had the best protein production yield; therefore, this strain was used for recombinant HpEno expression. Other conditions of overexpression, such as culture temperature (25, 30 or 37°C), induction time (2, 3, 4, or 6 hours or overnight), and IPTG concentration (0.5, 0.7 or 1 mM), were assayed. All attempts to produce soluble HpEno by modifying the expression conditions were unsuccessful. The optimum expression was achieved by incubating the strain Rosetta-gami at 37°C for four hours after the addition of 1 mM IPTG. However, the expressed protein was insoluble and precipitated in inclusion bodies (Figure 2(a)).

Solubilized histidine-tagged HpEno in 8 M urea (or 6M guanidinium chloride) was immobilized on a metal ion affinity chromatography column (Ni-NTAresin) to eliminate contaminant proteins. Refolded protein was obtained by the removal of urea or guanidinium chloride by dialysis.

It has been reported that His-tagged enolase is prone to aggregation and that its solubility considerably increases when the His-tag is removed [27]. Therefore, all His-tag removal tests were carried out at low concentrations of enolase (approximately 0.1 mg mL^−1^). For His-tag cleavage, several experimental conditions, such as incubation time, buffer and concentration of PSP, were assayed. The highest cleavage efficiency was 50%, probably due to the lack of accessibility to the target site because of enolase dimerization and/or aggregation [50] (Figure 2(b)). With these conditions, the purity of the isolated soluble protein was higher than 95%, and the yields were approximately 4-5 mg per litre of culture.

### 3.6. Secondary and Tertiary Structures of Recombinant apo- and holo- HpEno

**Figure 3:**
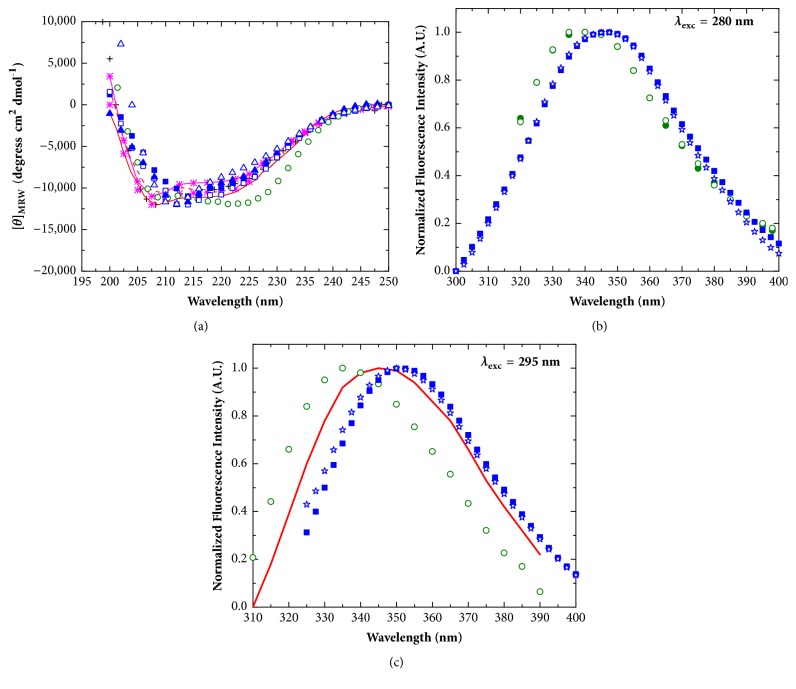
**Secondary and tertiary structure of enolases**. (a) Far-UV circular dichroism spectra; black cross:* Streptococcal surface *enolase; red line:* P. falciparum *enolase (dimer); red dashed line:* P. falciparum *enolase (monomer);pink line with asterisks:* S. pyogenes enolase* (TME buffer); pink asterisks:* S. pyogenes enolase* (Glycine buffer); green circles:* S. cerevisiae *enolase; blue closed squares: apo-HpEno (Tris-HCl); blue open squares: holo-HpEno (Tris-HCl); blue closed triangles: apo-HpEno (potassium phosphate); blue open triangles: holo-HpEno (potassium phosphate). (b) Fluorescence emission spectra for the excitation wavelength of 280 nm; Green closed circles:* S. cerevisiae *enolase (Tris-acetate); green open circles:* S. cerevisiae *enolase (Tris-HCl); blue closed squares: apo-HpEno(Tris-HCl); blue stars: apo-HpEno (Tris-acetate). (c) Fluorescence emission spectra for the excitation wavelength of 295 nm; red line:* P. falciparum *enolase; green open circle:* S. cerevisiae *enolase (Tris-HCl); blue closed squares: apo-HpEno(Tris-HCl); blue stars: apo-HpEno (Tris-acetate).

The secondary and tertiary structures of recombinant apo- and holo-HpEno were analysed by far-UV circular dichroism (CD) and fluorescence spectroscopies. In Figure 3(a), the CD spectra of HpEno are shown and compared with the spectra of enolases from other organisms. It is clear that the CD spectra of apo- and holo-HpEno obtained in different buffer conditions are almost super imposable. We did not observe any difference between spectra when urea was replaced by guanidinium chloride in the purification protocol (data not shown). These results mean that the secondary structure of the recombinant HpEno does not vary substantially with the buffer or with the presence of divalent cations. Additionally, these spectra resemble the spectra reported for other orthologs, implying that the secondary structure content is comparable to that of other enolases.

In Figures 3(b) and 3(c), the intrinsic fluorescence emission spectra of apo- and holo-HpEno are shown. The spectra of HpEno are red-shifted compared to the spectra from other species, indicating that the tryptophanyl residues in these proteins are more exposed to the solvent than the residues in the* Plasmodium falciparum* and* S. cerevisiae* enzymes are.

**Figure 4:**
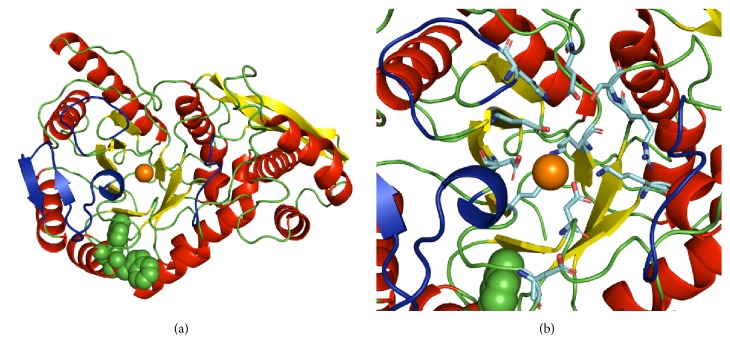
**Ribbon representation of the structure HpEnomonomer**. *β*-Strands are coloured in yellow, and *α*-helices are red. Tryptophans (W294 and W297) are shown as green spheres. The orange sphere represents a Mg^2+^ ion in the model that was obtained by SWISS-MODEL and rendered using PyMOL. Loops of the active site (L1, L2, and L3) are coloured in dark blue. In panel (b), the side chains of catalytic residues are shown.

Dozens of X-ray structures of enolase have been reported so far. This enzyme has been found to be either dimeric [51] or octameric [18, 52]. Each subunit of enolase is made up of two domains: the smaller N-terminal domain and the larger C-terminal domain. The N-terminal domain has a *β*_3_*α*_4_ topology. The second domain has the eightfold *βα* barrel structure with *ββαα*(*βα*)6 topology. In Figure 4, the ribbon representation of the HpEno structure was obtained by homology modelling using SWISS model [53]. The secondary structure has the characteristics of the archetypical scaffold of other enolases. Furthermore, the sequence of HpEno has two tryptophan residues (W294 and W297). The side chain of W294 is completely exposed, which can explain the red-shifting of the fluorescence spectrum. The same analyses were performed for enolases from* S. cerevisiae* and* P. falciparum*. In both cases, although these proteins have a larger number of tryptophanyl residues (five and four, respectively), their side chains seem to be less exposed to the solvent.

### 3.7. Biophysical Characterization

#### 3.7.1. Thermal Unfolding Profiles

**Figure 5:**
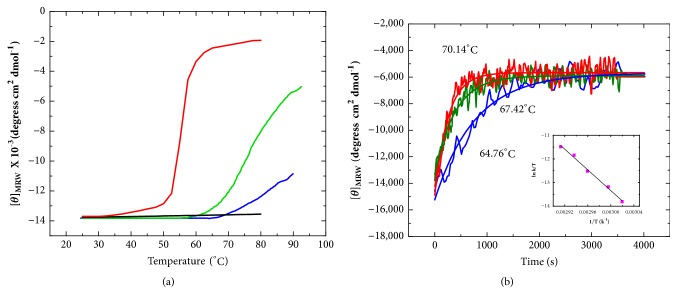
**Thermal unfolding of* H. pylori* enolase**. (a) Unfolding transitions of apo- and holoenolase from* H. pylori *monitored by CD spectroscopy at 220 nm; the experiments were carried out in 50 mMTris-HCl buffer at pH 7.4. Blue line: apo-HpEno, green line: holo-enolase (Mg^+^ 2 mM), black line: apo-ScEno, and red line: holo-ScEno (Mg^+^ 2 mM). (b) Kinetics of the thermal denaturation curves of holo-HpEno at different heating rates. The denaturation process was monitored by recording the ellipticity at 220 nm at a constant temperature. The insets correspond to the plots ofln(*k*_*1*_*/T*) versus 1/T, which represents the Eyring equation.

The thermal stability of apo- and holo-HpEno was analysed by temperature-induced denaturation scans. In Figure 5(a), the denaturation profiles of HpEno in Tris-HCl (50 mM) buffer at pH 8 are shown. The unfolding profiles of both apo- and holo-HpEno could not be monitored to completion because the melting temperatures were close to or even higher than the maximum temperature that can be attained by the Peltier accessory of our equipment. Therefore, the profiles obtained under these experimental conditions are useless for obtaining accurate thermodynamic parameters from the thermal denaturation. For comparison, the denaturation scans of yeast enolase were obtained under the same experimental conditions. It is noticeable that the HpEno transitions appeared to be approximately 25-30°C above the temperature for the transitions of yeast enolase. Additionally, the denaturation profiles of HpEno are less cooperative than those of yeast enolase. Cooperativity in thermal denaturation profiles is often regarded as an indication of native-like packing. Nevertheless, the formation of dimers or higher order oligomers can also contribute to cooperativity. We did not investigate the oligomeric state of any of the enolases at the denaturation temperatures, and sometimes, the dissociation of dimers cannot be detected in CD signals [54]. Therefore, we conclude that HpEno is a more stable protein than the enolases from other species, considering the unfolding temperatures, although the denaturation transitions of HpEno are less cooperative than those of yeast enolase.

Another important feature is the effect of Mg^2+^ on the thermal stability. Although this cation stabilizes yeast enolase, Mg^2+^ has the opposite impact on HpEno. These results were confirmed by the experiments performed in different buffer conditions (Tris-acetate, data not shown), with comparable results in all cases. Additionally, some reports indicate that Mg^2+^protectsagainst the imidazole-induced (or imidazole- + NaCl-induced) dissociation of rabbit muscle enolase and yeast enolase but does not have the same effect on* P. falciparum* enolase [6].

#### 3.7.2. Kinetics of Unfolding of Enolase

The time-course evolution of the temperature-induced unfolding reaction with* H. Pylori *holo-enolase was examined by far UV-CD at 220 nm in Tris–HCl buffer with 4 mM Mg^2+^. In Figure 5(b), some representative curves are shown. The kinetic curves show single-exponential decay profiles. Therefore, the curves were analysed using single-exponential decay equations (equation 1, supplementary materials: appendix 1). The rate constants obtained from each curve were used for the Eyring plot (inset of Figure 5(b)). From the slope of this plot, the activation enthalpy (ΔH^≠^) associated with the thermal unfolding of holo-HpEno was calculated (Table 2). In the same table, the corresponding ΔH^≠^ reported for yeast holoenolase [28] is also shown. Interestingly, both activation enthalpies are equivalent. This result means that the energy associated with the conformational changes between the native state of holo-HpEno and the transition state in the unfolding pathway is similar. It is worth mentioning that the kinetic curves of apo-HpEno were not studied because the thermal stability is too high, making it difficult to monitor under the experimental conditions used in this study.

### 3.8. Enzymatic Activity

**Figure 6:**
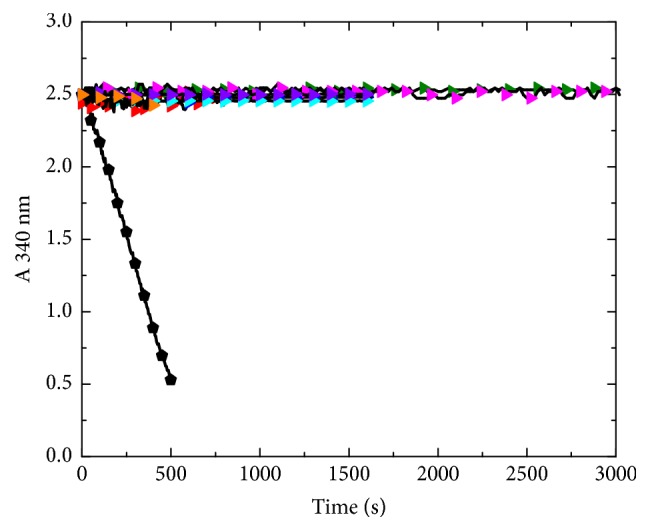
**Enzymatic Activity of HpEno**. Black circle: ScEno TEA buffer with MgSO_4_ (25 mM); red triangle: HpEno in tris-acetate buffer with MgSO_4_ (5 mM); green triangle: HpEno in TEA buffer with MgSO_4_ (5 mM); blue triangle: HpEno in HEPES buffer with MgSO_4_ (0.5 mM); cyan triangle: HpEno in TEA buffer with MgSO_4_ (0.5 mM); yellow triangle: HpEno with the His-tag in TEA buffer with MgSO_4_ (25 mM); pink triangle: crude extract from inclusion bodies in TEA buffer with MgSO_4_ (5 mM); orange triangle: HpEno in TEA buffer with MgSO_4_ (25 mM).

The enzymatic activity of recombinant* H. pylori* enolase was assessed following the standard protocol, as well as by exploring a variety of experimental conditions. Unexpectedly, the conversion of the substrate 2-PGA into PEP could not be detected (Figure 6), although we also evaluated other experimental conditions reported for enolases from other biological species. In addition, we tried the tests with cleaved or uncleaved enolase, different protein concentrations and experimental parameters, such as temperature, monitoring time, buffer solution and concentration of reactants. The inclusion bodies from crude extracts were also used in assays. All these attempts could not detect any substrate conversion, whereas the positive control showed enzymatic activity in a large variety of conditions. Furthermore, the control experiments for each set of assays showed that the auxiliary enzymes were not a limiting factor.

## 4. Discussion

The recombinant protein (HpEno) was overexpressed using the* E. coli *strain Rosetta-gami; nevertheless, under all conditions tested, the recombinant protein was obtained in the insoluble fraction (inclusion bodies). HpEno was solubilised using urea or guanidinium chloride and further purified to homogeneity by affinity chromatography, with yields of approximately 4-5 mg per litre of culture. The recombinant protein obtained in this way acquired the native-like secondary structure, resembling that of other enolases, as judged from the shape and intensity of the CD spectra.

The thermal denaturation transitions of apo- and holo-HpEno were monitored by far-UV circular dichroism (*λ*=220 nm). The profiles started at very high temperatures (approximately 70 and 75°C) and did not reach completion even at 90°C, which is the highest temperature attainable by our Cd equipment. It is clear that HpEno is much more stable than yeast enolase (Figure 5(a)), although the thermal stability cannot be established solely from melting temperatures, since several considerations must be taken into account, like reversibility of the unfolding transition and the denaturation mechanism. The high stability of HpEno implies that the recombinant protein has a well-established network of intramolecular contacts. It is important to point out that Mg^2+^ has a destabilizing effect in the case of HpEno, similar to the situation for the enolase from* P. Falciparum *[55], while the common trait is that cofactors stabilize enolase as well as many other enzymes [56].

The kinetics of the thermal denaturation reaction was also studied for holo-HpEno. The activation enthalpy associated with the unfolding process is almost equivalent between yeast enolase [28] and recombinant HpEno (obtained at similar experimental conditions). The activation enthalpy measures the disruption of enthalpically favourable interactions in the folded state that occur to achieve the transition state. The almost equal unfolding activation enthalpies for both proteins means that HpEno and yeast enolase might show a similar number (and character) of favourable interactions. This finding can also be considered the proof of a well-packed, native-like structure of recombinant HpEno. Indeed, HpEno is more stable than yeast enolase, which is not inconsistent with similar unfolding activation enthalpies, because there are several ways to increase the overall stability of the native state protein, such as decreasing the value of the equilibrium constant of denaturation or increasing the rate constant of renaturation. Alternatively, the native state can also be stabilized by reducing the rate constants of denaturation. One or more of these possibilities thereof would favour the native state. Furthermore, the activation enthalpy is not calculated from the absolute value of the rate constants of denaturation but from their dependence on temperature. Therefore, two proteins with different conformational stabilities can show similar activation enthalpies for thermal unfolding [28].

In this work, several experimental conditions were used to assess the enzymatic activity of HpEno; nevertheless, none of the conditions was successful. Several plausible explanations for this result can be proposed. The most immediate is incorrect folding of the recombinant protein. The secondary structure of HpEno, like that observed for the enolase from other biological species, and the high thermal stability of HpEno are not compatible results with the lack of activity. Neither, the activation enthalpy is associated with denaturation, which is an indicative of similar native contacts when compared to other enolases. Therefore, the incorrect folding of the protein HpEno should not be the most plausible cause for the lack of activity. Some reports indicated that enolases from* X. fastidiosa* and chicken muscle and liver were irreversibly denatured by urea, suggesting that this chaotropic agent should not be used to extract enolase from inclusion bodies [57]. In contrast, we have found 85-90% reversibility of the urea-induced unfolding reaction for yeast enolase [58]. When guanidinium chloride was used in the isolation protocol instead of urea, we observed similar results. In addition, the absence of enzymatic activity cannot be attributed to the absence of catalytic and metal-binding sites residues within HpEno since the sequence analysis indicated that both sites are well conserved in HpEno. Therefore, this result indicates that some amino acid changes in the sequence different from those at the active sites for glycolysis could modify the structure of functional domains. At this point, it is not possible to conclude convincingly the lack of glycolytic activity of HpEno, although abundant experimental evidence showed the dearth. Indeed, proving lack of activity of an enzyme is much more difficult than confirming one. Therefore, a much wider range of experimental conditions.

Interestingly, in the genome of* H. pylori* 26695, genes exist for most enzymes in the glycolysis pathway, except for the following three genes:* pyk*, pyruvate kinase, the enzyme corresponding to the last step for the formation of pyruvate in glycolysis (exactly following the enolase functional step);* pfk*, phosphofructokinase; and* gnd*, 6-phosphogluconate dehydrogenase. In addition, the activity of phosphoglycerate mutase (*pgm*), the enzyme catalysing the step immediately preceding the enolase reaction in glycolysis, has not been detected [59]. Thus, in the strain 26695, the reaction catalysed by enolase might not be necessary for glycolysis to produce pyruvate. Compared with the more than 100* H. pylori* genomes sequenced, the genome of this strain also provides a similar result. The gene* pyk* exists in some strains of* H. pylori* but without the genes* pfk* and* gnd*. The gene* ppsA* responsible for the reverse reaction of pyruvate kinase, called phosphoenolpyruvate synthase, exists in all of the strains. On the other hand, it was found that glucose oxidation took place via the Entner-Doudoroff pathway [60], which was confirmed by the genomic analysis showing that the entire Entner-Doudoroff pathway exists in* H. pylori *[61]. From previous results from our lab, the expression of HpEno has been detected in the cell lysate of* H. pylori* 26695. Its presence was observed in the pull-down products using the protein MreB as a bait [22].

If the lack of activity of HpEno is confirmed, considering that enolase is indeed expressed in* H. pylori, *an important question emerges: what would be the function of this protein in this organism? Enolase is a protein whose moonlighting features have been well established in several organisms [1]. In prokaryotic organisms in particular, enolase has been found on the cell surface acting as a plasminogen-binding protein (*S. pneumoniae*). In the same organism, a host-derived exRNA-binding protein has recently been described [49]. Both noncanonical functions of enolase have been proposed as possible virulence factors of this pathogen. It is worth mentioning that HpEno has been found to be enriched in the extracellular proteomes of* H. Pylori *[36], indicating its probable participation in moonlighting functions. Sequence analysis indicated the presence of a similar plasminogen-binding site, which cannot be confirmed since this site lacks some of the positive residues commonly found in other plasminogen-binding enolases. Another possibility is that this protein could bind host-derived exRNAs, as indicated by the presence of several conserved residues of the putative binding site in HpEno [49]. These moonlighting activities need to be clarified by further in-depth studies.

## 5. Conclusions

The enolase protein is well conserved from bacteria to humans, even in the smallest free-living bacterium,* Mycoplasma genitalium* G37 (515 individual genes), indicating that the gene should have an important function in cells. The basic function of enolase is its role as a key enzyme in the production of pyruvate via glycolysis. On the other hand, enolase is also a moonlighting protein, and many new functions have been found in recent years. Enolase is a good target for new drug discoveries for the control of pathogen infection.* H. pylori* is a widespread bacterial pathogen in humans with resistance to multiple antibiotics, leading to difficult treatment. The characteristics of HpEno are the basis for understanding the exact function in this particular bacterium. Analysis of the amino acid sequence of this protein compared with the sequences from other species showed conservation of the active sites of glycolysis. After purification of recombinant HpEno, the secondary structure of HpEno was also determined; HpEno generally showed similar features as the homologues from other species but still had distinct traits in some respects. It was not possible for us to detect any enzymatic activity; nevertheless, defining conclusively the lack of function in the glycolytic pathway is a challenge requiring further study. In addition, more experimentation should be carried out to determine other probable functions of HpEno in this pathogen. This information could be useful in proposing novel strategies to fight* H. pylori* infection.

## Figures and Tables

**Table 1:** Amino acids involved in enzymatic activity and biological functions of enolases.

**Feature**	***S. cerevisiae***	***H. sapiens***	***E. histolytica***	***S. pneumoniae***	***H. pylori***	***X. fastidiosa***
**Mg** ^**2+**^	D^247^ E^296^ D^321^	S^40^ D^245^ E^293^ D^318^		D^242^ E^291^ D^318^	D^242^ E^286^ D^313^	D^244^ E^287^ D^314^

**Catalytic sites**	H^160^ E^169^ E^212^ E^296^ D^321^ K^346^ R^375^ S^376^ K^397^	H^158^ E^167^ E^210^ E^293^ D^318^ K^343^ R^372^ S^373^ K^394^	H^156^ E^165^ E^208^ E^296^ D^322^ K^347^ R^376^ S^377^ K^398^	H^155^ E^164^ E^205^ E^291^ D^318^ K^343^ R^372^ S^373^ K^394^	H^155^ E^164^ E^205^ E^286^ D^313^ K^338^ R^367^ S^368^ K^389^	H^157^ E^166^ E^207^ E^287^ D^314^ K^339^ R^368^ S^369^ K^390^

**Loops of active site**	^37^SGASTGVH^44^ ^159^SHAGGALA^166^ ^250^SSEFFKDGKYDLD FKNPESDKSKWL^274^	^37^SGASTGIY^44^ ^157^SHAGNKLA^164^ ^248^ASEFFRSGKYDLD FKSPDDPSRYI^271^	^37^SGASTGVH^44^ ^155^AHAGNALA^162^ ^246^ASEFYNEETKKYD LGKKIPADKKDPSLVK^274^	^39^SGASTGEH^46^ ^154^SHSDAPIA^161^ ^245^SSEFYDKERKVYD YTKFEGEGAAVR^269^	^39^SGASTGKR^46^ ^154^THANNSID^161^ ^245^SSELVDENFNYHL KGENKIL^264^	^39^SGASTGTK^46^ ^156^AHADNNVD^163^ ^247^SSEFRDNGKYNLV GENKRL^266^

**RNA binding**	^56^KWMGKGVLH^64^ ^103^KSKLGA^108^ ^192^HNLKSLTKKRYGA SAGN^208^ ^315^GIQI^318^ ^403^RSERLAK^409^ ^435^DKL^437^	^56^RYMGKGVSK^64^ ^103^KSKFGA^108^ ^190^HNLKNVIKEKYGKD ATN^206^ ^312^GIQV^315^ ^400^RSERLAK^406^ ^432^LAK^434^	^56^RYGGKGVLK^64^ ^101^KGKLGA^106^ ^188^QCLKVVIKAKYGQ DATN^204^ ^315^GNFQI^319^ ^404^RSERLCK^410^ ^435^TA^436^	^58^RYGGLGTQK^66^ ^103^KGKLGA^108^ ^187^HALKKILKSRGLET A^201^ ^310^GKKVQL^315^ ^400^RTDRIAK^406^ ^432^LKK^434^	^58^RFLGKGVLR^66^ ^103^YANLGA^108^ ^187^HTLKKLLDGKNQLT^200^ ^305^GRQIQL^310^ ^395^RSERIAK^401^ ^420^G^420^	^58^RYLGKGVRA^66^ ^103^KGRLGA^108^ ^189^HALKSVLKGQGLST^202^ ^306^GHKVQL^311^ ^396^RSDRVAK^402^ ^428^LKS^430^

**Enolase signature**	^343^LLLKVNQIGTLSES^356^	^340^LLLKVNQIGSVTES^353^	^344^VLIKVNQIGTLTET^357^	^340^ILIKVNQIGTLTET^353^	^335^VLIKPNQIGTISET^348^	^336^ILIKLNQIGTLTET^349^

**Hydrophobic domain**	^33^SIVPSGAST^41^	^33^AAVPSGAST^41^	^33^SCVPSGAST^41^	^35^GMVPSGAST^43^	^35^AIVPSGAST^43^	^35^AAVPSGAST^43^

**Plasminogen binding**	^253^FFKDGKY^259^	^251^FFRSGKY^257^	^249^FYNEETKKY^257^	^248^FYDKERKVY^256^	^248^LVDENFNY^255^	^250^FRDNGKY^256^

**Table 2:** Activation enthalpies associated to the thermal unfolding reaction of enolases.

**Organism**	**Condition**	**Proposed mechanism**	Δ**H**^≠^**(kJ mol**^**−1**^**)**
***H. pylori***	+Mg^2+^	$N_{2}\overset{k}{\to }2D$	179 (8)

**Yeast**	+Mg^2+^	$N_{2}\overset{k}{\to }2D$	190 (30)

## Data Availability

The data used to support the findings of this study are available from the corresponding author upon request.

## Supplementary Materials

Supplementary Materials Appendix 1. File containing extended methodological details.
