# Supplementary material for: Biochemical and Biophysical Characterization of the Enolase from Helicobacter pylori
Source: Biomed Res Int. 2018 Dec 17;2018:9538193. doi: 10.1155/2018/9538193 (PMC6311853; doi:10.1155/2018/9538193)
Supplement: Supplementary Materials — Appendix 1. File containing extended methodological details. [file 9538193.f1.docx]

## SUPPLEMENTARY APPENDIX 1

## Methodological details

### Activity assays

The coupled reactions of rabbit muscle pyruvate kinase (PYK) and beef heart lactate dehydrogenase (LDH) were used to test enolase activity by following the decrease of NADH absorbance at 340 nm using a Scinco S-3100 UV-Vis spectrophotometer. Standard activity assays were performed at 25 °C in a 1.0 mL reaction mixture containing 81 mM triethanolamine/HCl, pH 7.6, 1.9 mM 2-PGA, 1.3 mMADP, 0.12 mMß-NADH, 0.5 mMMgSO_4_ and 25 mMKCl. The auxiliary enzymes PYK and LDH were used at final activities of 7 and 10 U·mL^−1^, respectively, and were purchased from Sigma. Recombinant enolase was added at a final concentration of 400 ng mL^-1^.

### Protein purification from inclusion bodies

The pellet was suspended in 50 mL of buffer A (50mM Tris-HCl pH 8.0, 1 mM EDTA, 150 mMKCl, 0.1 mM PMSF) and disrupted by sonication with pulse set for 10 s followed by 50 s rest at an amplitude of 50 %, for total 1 min of sonication time. After sonication, 10 mM MgSO_4_was added to chelate the EDTA and lysozyme in about 0.1 mg mL^-1^ to the lysate. The solution was incubated at room temperature (RT) for 20 min and centrifuged to collect inclusion bodies for 20 min at 9,000 rpm and 4°C. The pellet was re-suspended in 50 mL of buffer B (50 mMTris-HCl pH 8.0,1 % Triton X-100 v/v, 1 mM EDTA, 0.1 mM PMSF), then inclusion bodies were sonicated again, another portion of MgSO_4_ and lysozyme was added and incubated at RT for 20 min. The solution was centrifuged, and the pellet was washed out with 50 ml of buffer C (50 mMTris-HCl, pH 8.0, 1% Triton X-100 v/v, and 0.1 mM PMSF), incubated at RT for 20 min and centrifuged. A final wash was performed suspending the pellet in 50 mL of buffer D (deionized water complemented by 0.1 mM PMSF) and incubated at RT for 20 min and centrifuged with 9,000 rpm at 4 °C. The pellet was then suspended in 50 mL of solution containing 8 M urea and 1 mM PMSF, incubated at RT for 4 hrs. This solution was centrifuged, and the supernatant was stored at 4°C.

### Thermal denaturation transitions monitored by circular dichroism

Thermal denaturation transitions were carried as described out by increasing the temperature of the samples from 25 to 95 °C at constant heating rates of 2 ºC min^-1^, while changes on the ellipticity at 220 nm were monitored. Cooling profiles were recorded after denaturation transitions had gone to completion. The temperatures of the sample within the cell were registered with the external cell holder probe. Enolase solutions (HpEno) prepared at a concentration of 0.060 mg mL^-1^. The assays were accomplished in three different buffers: Tris-acetate, Tris-HCl and potassium phosphate, all of which are at pH 7.4 and 50 mM.In some experiments, solutions were complemented with 2 mM MgCl_2_, or 1 mM EDTA as stated.

Since the denaturation transitions of HpEno do not show the appearance of detectable intermediates, these data were analysed using the two-state denaturation model for a dimeric protein, where the only species detectable are the Native dimer (N_2_) and the unfolded monomers (D), according to:

(1)

In this case, the equilibrium constant *K_D_* is defined as:

$K_{D}=\frac{[D]^{2}}{[N_{2}]}$ (2)

and the total protein concentration in terms of monomer, *P_t_*, is $P_{t}=2[N_{2}]+[D]$.

The fraction of monomers involved in native dimers,$f_{N_{2}}$, is given as $f_{N_{2}}$*=1-f_D_*, where *f_D_* is the fraction of unfolded monomers, and

$f_{N_{2}}=\frac{2[N_{2}]}{P_{t}}$ (3)

$f_{D}=\frac{[D]}{P_{t}}$ (4)

Therefore,

$K_{D}=\frac{[D]^{2}}{[N_{2}]}=2P_{t}\frac{f_{D}^{2}}{f_{N}}$ (5)

The equilibrium constant could be expressed as a function of temperature. Outside the transition region the CD signal of the native and denatured species varies linearly with temperature; therefore, $f_{N_{2}}$ and $f_{D}$ were estimated by extrapolation at each temperature. The enthalpy of denaturation (*ΔH_D_*) was obtained by plotting ln *K*_D_ versus *T* according to the van’t Hoff equation:

|  | (6) |
| --- | --- |

where *T_m_*is the midpoint of thermal denaturation. The van't Hoff plots (ln *K_D_* vs *1/T*) of thermal denaturation are approximately linear through the range of temperatures studied, allowing an estimation of the enthalpy of unfolding at T_m_.

### Denaturation Kinetics

The kinetic curves of HpEno were obtained by recording changes in ellipticity at 220 nm. The 1.0 cm path-length cell was filled up to at least 98% of its total volume (3.0 mL) with 100 mM tris HCl buffer complemented with 4 mM MgSO_4_, and the solution was equilibrated at the temperature of each test. Afterwards, the necessary volume (maximum 2 % of the total volume) of concentrated enolase solution was added to the cell, and the CD signal was registered immediately. Samples were vigorously stirred to promote rapid mixing and temperature equilibration. Under these conditions, the dead time of experiments was less than ten seconds.

Kinetic curve data were fitted to single exponential decay equations (*θt = θf + A1 exp* [*–k_1_t*]); where *θt* is the CD signal measured at time *t*, *θf* is the final CD signal, value *A_1_* represents the amplitude of the exponetial curve, and, *k_1_* is the unfolding rate constant of the reaction*.* The dependence of the rate constants on the temperature was assesed by plotting the ln(*k_1_/T*) versus 1/T, which represents the Eyring´s equation.

(7)

where *k_B_* (1.3807 X 10^-23^ JK^-1^) and *ħ* (6.62 X 10^-34^ J) are Boltzman and Planck constants respectively, *ΔS^‡^* and *ΔH^‡^* are the entropy and enthalpy of activation respectively.
